# An equitable and sustainable community of practice framework to address the use of artificial intelligence for global health workforce training

**DOI:** 10.1186/s12960-023-00833-5

**Published:** 2023-06-13

**Authors:** Seble Frehywot, Yianna Vovides

**Affiliations:** 1 Department of Global Health & Health Policy and Co-Founder of IT for Health and Education System Equity, George Washington University Milken Institute of Public Health, Washington, DC USA; 2 Senior Director of Learning Design and Research at the Center for New Designs in Learning and Scholarship (CNDLS), Curriculum Director for the Learning, Design, and Technology (LDT) program, and Co-Founder of IT for Health and Education System Equity, Georgetown University, Washington, DC USA

**Keywords:** Artificial Intelligence, Community of practice, Health workforce training, Equity; Machine Learning, Capacity-building

## Abstract

Artificial Intelligence (AI) technologies and data science models may hold potential for enabling an understanding of global health inequities and support decision-making related toward possible interventions. However, AI inputs should not perpetuate the biases and structural issues within our global societies that have created various health inequities. We need AI to be able to ‘see’ the full context of what it is meant to learn. AI trained with biased data produces biased outputs and providing health workforce training with such outputs further contributes to the buildup of biases and structural inequities. The accelerating and intricately evolving technology and digitalization will influence the education and practice of health care workers. Before we invest in utilizing AI in health workforce training globally, it is important to make sure that multiple stakeholders from the global arena are included in the conversation to address the need for training in ‘AI *and* the role of AI in training’. This is a daunting task for any one entity and a multi-sectorial interactions and solutions are needed. We believe that partnerships among various national, regional, and global stakeholders involved directly or indirectly with health workforce training ranging to name a few, from public health & clinical science training institutions, computer science, learning design, data science, technology companies, social scientists, law, and AI ethicists, need to be developed in ways that enable the formation of an equitable and sustainable Communities of Practice (CoP) to address the use of AI for global health workforce training. This paper has laid out a framework for such CoP.

## Introduction

In 1950, the English mathematician Alan Turing wrote a landmark paper titled “Computing Machinery and Intelligence” posing the question: “Can machines think?” This led to further exploration of the use of machines to support human decision-making with a 1956 workshop, organized by John McCarthy, an American mathematician, that focused on the “study of artificial intelligence.” [1] The field was born. Yet, more than a half-century later, we are still struggling with a definition; artificial intelligence (AI) is most often being defined more in “what its *capabilities* are rather than strictly defining what it *is.”* [2] In this commentary, we will use McCarthy’s definition that describes AI as “the science and engineering of making intelligent machines, through algorithms or a set of rules, which the machine follows to mimic human cognitive functions, such as learning and problem solving” [3] as this definition provides a blend of both its capabilities and what it is. Within healthcare, AI applications such as image processing for improved cancer detection, that have been created are being refined using computer science techniques such as machine learning. Machine learning (ML) uses mathematical data models or algorithms to “mimic” human reasoning and therefore relies on multiple algorithmic approaches such as neural networks, deep learning, natural language processing, and others. [4]

This commentary is *a call to action* for engaging in a global community of discourse and innovation to address health workforce training so that AI technology is not the driver but rather the support for human decision-making. We need AI to be able to ‘see’ the full context of what it is meant to learn. AI trained with biased data produces biased outputs and providing health workforce training with such outputs further contributes to the buildup of biases and structural inequities. AI-based tools have “the potential to worsen health inequities if implemented without deliberate action to ensure fairness and there is a need for all clinicians to understand the social, ethical, legal, and regulatory issues that will determine whether AI-based tools will narrow or widen health disparities and health care gaps.” [5] The pandemic left the world without much choice in the adoption of new technologies; however, given that now there is a choice, it is imperative that we consider the adoption of technologies in global health workforce training, especially AI, with care and ask, ‘who are we leaving behind?’ and ‘what can we do about it to mitigate the risk of widening health disparities?’ As Alondra Nelson at the Institute for Advanced Study, Princeton University, stated, “We need to be honest about the past and how science and technology have harmed some communities, left out communities, and left people out of doing the work of science and technology." [6] Before we invest in utilizing AI in health workforce training globally it is important to make sure that multiple stakeholders from the global arena are included in the conversation to address “the need for training in AI *and* the role of AI in training.”[7]

## Why is a collaboration among various stakeholders needed in the use of Artificial Intelligence for global health workforce training?

The accelerating and intricately evolving AI technology and digitalization has and will influence the education and practice of health care workers. Therefore, there is the urgency of need to organize and collaborate among the various local, regional, national, and global stakeholders involved directly or indirectly with health workforce training.

When addressing AI for global health workforce training, we seek to provide a framework that enables the global health workforce to enter into the conversation on the use of AI in supporting human decision-making. The proposed framework addresses “the duality that health professions educators must consider now: the need for training in AI *and* the role of AI in training.” [7] We believe that partnerships among local, regional, national, and global stakeholders involved directly or indirectly with health workforce training, ranging to name a few, from public health & clinical science training institutions, computer science, learning design, data science, technology companies, social scientists, law, and AI ethicists, need to be developed in ways that enable the formation of equitable and sustainable Communities of Practice (CoP). CoPs are “groups of people who share a concern or an interest for something they do and, as they regularly interact, they learn to do it better.” [8]

Given that AI advances influence most disciplines, it is important to ensure that a CoP brings the diversity of voices for peer-to-peer learning to be meaningful. Having an established CoP could address crosscutting challenges. For example, if a medical school wanted to explore how to introduce AI into the medical education curriculum, the research team could include computer scientists, ethicists, medical education specialists, healthcare practitioners, students, and others. Through such collaboration, the team could research ways that AI could be taught in medical school to develop decision-making skills related to AI-supported applications.

Another team might be interested to examine human bias in machine learning in relation to the use of AI in healthcare. Instead of only having data science-focused discussions, a transdisciplinary team could be formed from the CoP members that bring data scientists, ethicists, social scientists, clinicians, and other stakeholders together to examine the sources of bias in datasets and AI systems through societal, structural, and technical perspectives so as to identify harm and impact. This would allow for an elevated discussion on how to design, use, and/or question the AI-powered systems with an equity lens ideally before adoption of AI in health workforce education.

CoP at the local, national, regional, and global levels across the professions is needed. We believe that the proposed CoP model, when applied with intentionality, can enhance knowledge about AI and allow voices from across the globe to guide the conversation based on their own contextual needs.

## An equitable and sustainable community of practice framework to address the use of Artificial Intelligence for global health workforce training

**Fig. 1:**
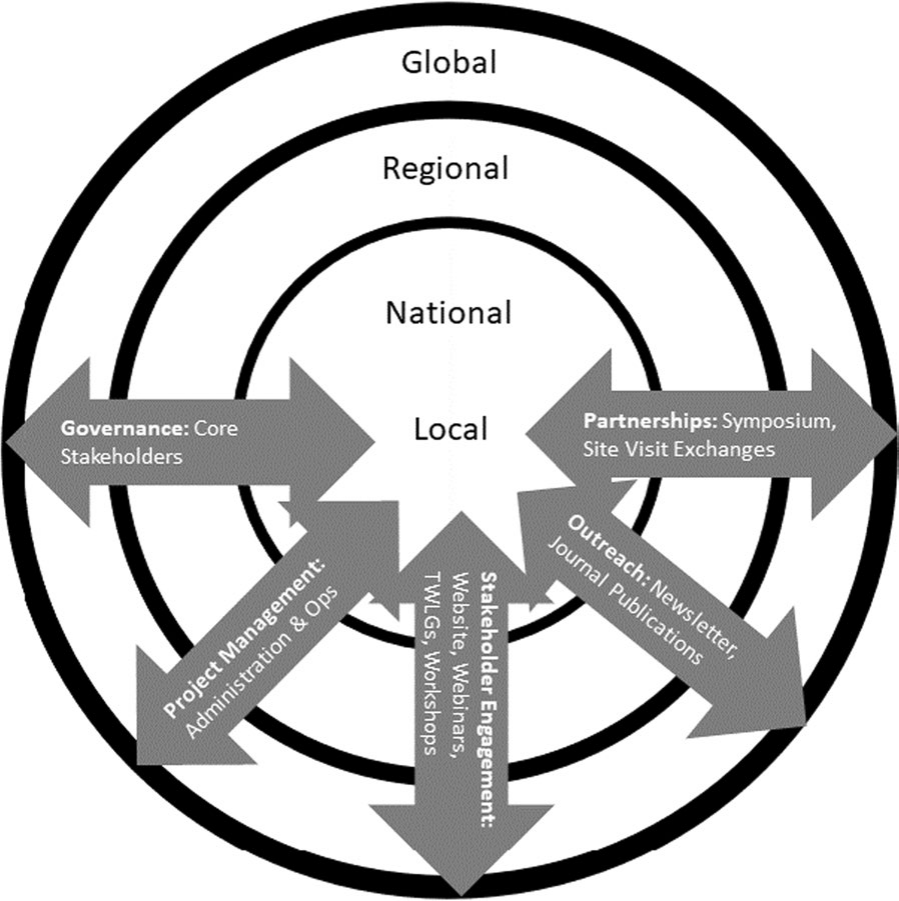
Framework of CoP: context, pillars, and components

Based on our experience in developing global health workforce-related CoPs [9], we envision that a sustainable and equitable CoP needs to address five structural pillars: **governance, project management**, **partnership**, **stakeholder engagement,** and **outreach** to enhance cross-fertilization of AI knowledge and adaptations of new AI knowledge among local, national, regional, and global stakeholders. The following seven components need to be in place to ensure that the pillars remain standing: (1) building and interconnecting interdisciplinary, stakeholder-initiated Technical Working-Learning Groups (TWLGs); (2) nurturing in-depth Exchanges of key stakeholders; (3) hosting an Annual Event for cross-fertilization of ideas among stakeholders; (4) promoting scholarship and partnerships with leading journals; (5) developing a website, to serve as a ‘digital town center’ of the CoP; (6) producing and distributing a Newsletter, and (7) hosting monthly transdisciplinary webinars and workshops that address AI-related topics that inform and educate from basic to advanced topics in relation to AI and health workforce training to ultimately support health equity. Figure 1 shows the connections among the broader context, the pillars, and the components.

The following descriptions provide a brief explanation as to the need for each pillar. In addition, we propose a set of components that could be conducted to advance the knowledge about AI within the health workforce training space. We are sharing this level of detail as part of our call to action to note that it is critical to formalize such an effort so as to demonstrate the breadth and depth of the reach of the CoP’s activities. Intentionality in designing the interactions across all CoP components among all stakeholders is key to the collaborative nature of the proposed effort.

1. **Governance**: includes recruiting, coordinating, and establishing a consortium of institutions and organizations that have a core mission to develop an equitable and sustainable AI CoP to advance knowledge of AI for informing health workforce training and also that aims to include under-represented or underserved groups impacted by health disparities. To enable smooth coordination and communication across the CoP, we propose an agreed upon timeline governance meeting that will aid in establishing an organizational structure for the CoP. At the local, national, regional, and global levels, there is a need for the presence of a coordinating entity that will carry out the duties of the governance pillar. This coordinating center can be situated within the Ministries of Health and Education, or in an international, regional, and local health and education governance entity.
2. **Project management**: The aim of this pillar is to address the overall goal of the CoP, to establish an organizational and project management structure, and to create a system that is visible for tracking the CoP activities across all input activities, output areas, and coordinating efforts. This system will help streamline and track the various milestones. Regular reporting of the status of activities across all CoP efforts would need to be shared. It is essential to maintain an agile approach in project planning and implementation to be responsive to external and internal requests, as needed. The project management team would coordinate and manage the integration of the CoP work activities among CoP participants and leverage the strengths and synergies of the CoP.
3. **Partnerships**: The aims of this pillar are multiple.
  To establish trusted relationships with health disparities groups, educators, computer, and data scientists to examine the data used in AI research and applications as well as how machine learning models are constructed.
  To build capacity among partners for seizing opportunities that catalyze rapid advancements in AI capabilities within their own contexts.
  To ensure coordination among the different activities, many of which will be interdependent, while at the same time balancing the need for a deliberate and strategic approach to building lasting partnerships and infrastructure.

To address the partnership goals, we have integrated three components: an Annual Symposium, a Site Visit Exchange, and a capacity-building program on Algorithmic Literacy that offers continuing education credits.

- *Annual symposium*
  Conducting an annual symposium will be highly valuable for fostering partnership among all CoP stakeholders. This will be a venue where the stakeholders will present findings, share ideas, learn from each other, and expand their knowledge. A steering committee that includes a CoP representative of each stakeholder will set the theme and components of the annual symposium. The role of the steering committee will be to provide balance and transparency to the various participant groups while building a substantive program.
- *Site visit exchange*
  CoP members need to conduct site visit exchanges to connect with peers among the institutions involved in the CoP. These sites visit exchanges aim to cross-fertilize ideas, and identify gap areas for research to move the pendulum for equitable and sustainable AI in global health workforce training toward tangible positive outcomes.
- *Algorithmic literacy program*
  This could be an online program that offers those taking it the opportunity for continuing education credits. It would be designed with a storytelling approach to help those participating explore AI in relation to healthcare applications. Topics could include, but are not limited to, human bias and AI, human and machine learning. CoP members could form a transdisciplinary team to develop each topic.

4. **Stakeholder engagement**: The aim of this pillar area is to establish and sustain meaningful virtual and in-person learning opportunities to engage all CoP stakeholders, ensuring coordination among the different activities, many of which will be interdependent, and building lasting relationships among stakeholders that can serve as a sustainable CoP. We have integrated three components: Town-Center (website), Technical Working-Learning Groups (TWLGs), and Monthly Webinars and Workshops.

- *Town-center (CoP website)*
  The CoP website serves as a “digital town square” for the full CoP community and for potential collaborators and stakeholders. Its home page could feature among many things “Noteworthy News” and “Noteworthy Events,” video clips of CoP stories. CoP webinars will also be featured. It could contain a library page (repository) on AI in health workforce education, AI ethics, data science, and other relevant topics based on an initial survey and needs of the CoP stakeholders.

- *Technical Working-Learning Groups (TWLGs)*
  By working collaboratively across all CoP stakeholders, the aim is for the CoP, to identify and establish TWLGs that can meet and dig deeper into a pertinent topic area such as AI Ethics, Health Equity, Data Research Techniques, AI applications, and others. The TWLGS will be led by members from the CoP who have an interest in that specific area. Each of the TWLG activities is determined by TWLG members.

- *Hosting regular webinars and workshops*
  After identifying the learning gaps of the CoP, there will be a need to conduct regular webinars and workshops with synchronous and asynchronous discussion led by peers.

5. **Outreach**: The aim of this pillar is to enhance connections among CoP stakeholders and to welcome new ones by sharing the work that CoP members are doing more broadly. This is a critical pillar as it is a path toward sustainability of efforts and reach. We have integrated two components: a Newsletter and a Special Journal Supplement as well as provisions for presentations at research conferences. In addition to these two components, the annual symposium also plays a key role in bringing in other vital stakeholders.

- *CoP newsletter*
  One of the mandates of the CoP is to construct a communication platform to facilitate engagement among the CoP consortium. A steering committee composed of the various CoP members could outline the contents and dissemination of the Newsletter. The newsletter could highlight a topic tackled by the CoP consortium members, serves the TWLGs for the sharing of tools, literature, and best practices, and also highlights articles that show the breadth and depth of the CoP efforts. This is a space where all voices can be included.

- *CoP-wide publication in a leading journal as a journal supplement*
  This component expands the work of the CoP members into scholarship. It would disseminate the knowledge created through CoP efforts focusing on the transdisciplinary nature of the work. It is customary for various disciplines to publish papers in journals that cater to the outputs of their respective disciplines. Since this CoP is an amalgamation of various disciplines, creating sustainable and equitable opportunities for sharing will need to traverse many discipline areas. Thus, this gives an opportunity to publish in various discipline journals such as suggested are IEEE, Human Resources for Health, Health Equity journal, etc., but the exact journal will be decided collaboratively with the CoP members.

The following table (Table 1) can be used as a checklist for those interested in supporting such efforts for advancing knowledge of AI for global health workforce training. It includes key outcomes based on the five pillars and is shared here as an example of how operations and outcomes can be used as a way to make the process transparent among key stakeholders.

**Table 1:** Example of operational blueprint of outcomes for structuring CoPs

Process outcomes
Establishing governance structure
Placing processes of communication with the governance team and key stakeholders
Placing mechanisms in place for recruiting CoP members
Recruiting CoP members
Implementing project management system with appropriate access for different stakeholders
Establishing process flow in relation to coordination
Conducting periodical symposia successfully to include all CoP institutions and organizations
Conducting site visits to establish connections for future pilots, research, and collaboration among members
Establishing program that offer certification/credits in algorithmic literacy
Establishing social network mapping analysis
Establishing web resources that are geared toward capacity building and knowledge sharing
Implementing webinars and/or workshops with synchronous and asynchronous discussions
Establishing TWLGs to address health workforce training with AI
Tracking reports and publications generated across CoP members
Highlighting articles that show the breadth and depth of the CoP efforts and disseminating the collective knowledge created
Publishing a journal supplement of the CoP works

**Fig. 2:**
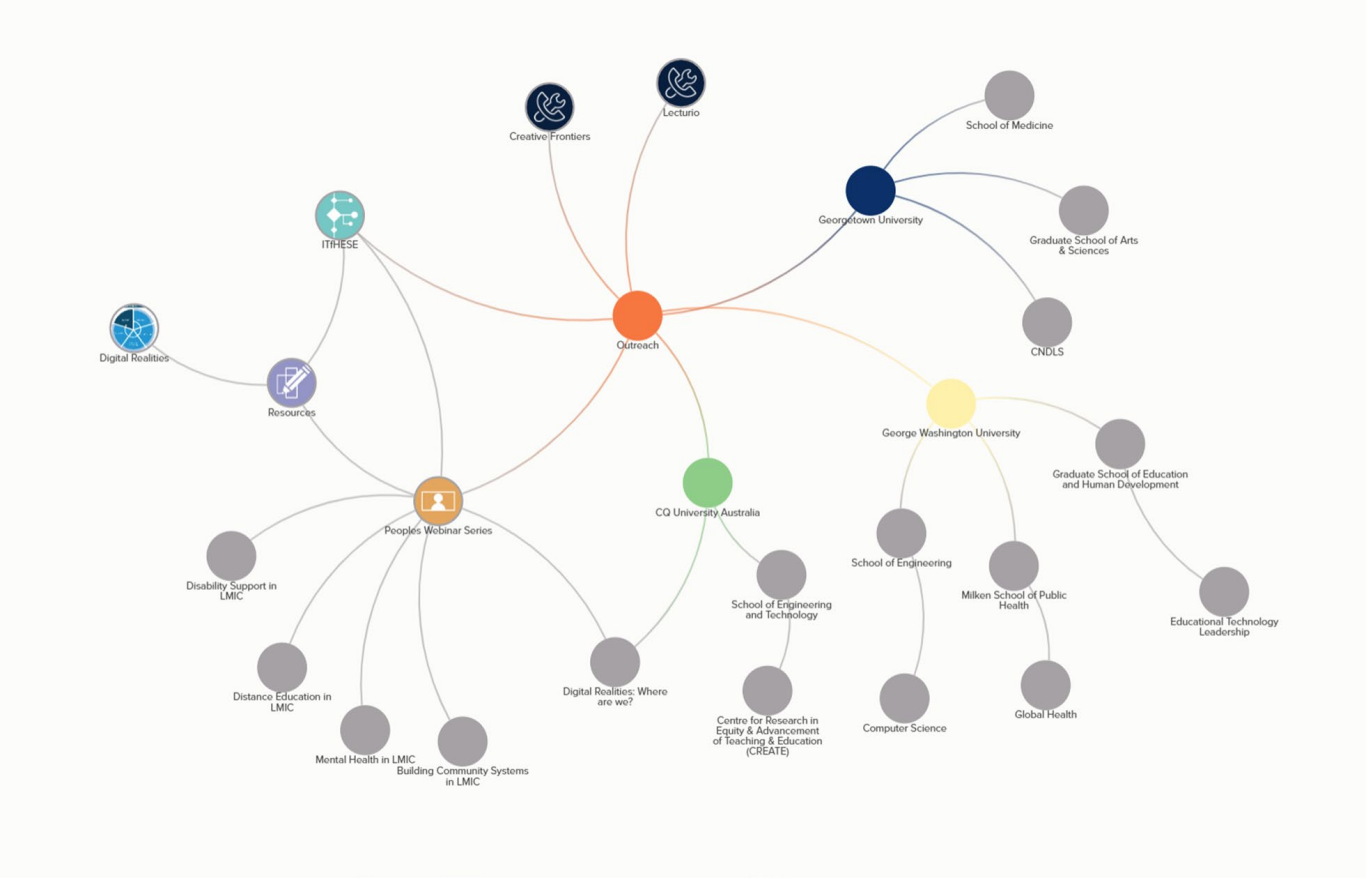
Example of Social Network Mapping of how we can represent the CoP members. SOURCE: IT for Health and Education System Equity Initiative (ITfHESE)

The CoP framework described in this commentary offers us a way to humanize artificial intelligence and machine learning by understanding what is behind the scenes of an application or a set of recommendations. This allows us to put those we serve in front. A transdisciplinary CoP has the potential to offer a stable foundation to address health equity issues within and across boundaries and borders. The links formed across the CoP members will strengthen the recruitment of new members and we believe will serve to promote transdisciplinary research teams. To ensure visibility of the efforts across the various connections, we recommend using Social Network Mapping and Analysis to show and understand the connections that will be forming across the various activities. Figure 2 shows an example of how such a diagram could serve both as a research and a communication tool.

## Conclusion

As stated in the Introduction***,*** this commentary ***is a call to action*** for engaging in a global community of discourse and innovation to address health workforce training so that AI technology is not the driver but rather the support for human decision-making. Currently, across the national, regional, and global arenas, the need for capacity building among health workforce education entities to address the risks and opportunities related to the advancement of AI technologies is palpable*. Therefore, there is the urgency of need to organize and collaborate among the various local, regional, national, and global stakeholders involved directly or indirectly with health workforce training.*

We have offered a framework for the creation of a transdisciplinary community of practice (CoPs) that focuses on artificial intelligence in health workforce training. The CoP framework offers us the opportunity to tackle this challenge from various disciplinary lenses. It enables us to learn to speak each other’s language. We might find that algorithmic literacy is not simply a continuing education approach but a core competency across all disciplines. The proposed CoP framework we propose has been tested within medical education and it works because of its simplicity and above all because of its inclusivity.

Artificial intelligence is propagating and, if embraced, it can be useful within health and public health workforce expertise areas; however, if we, as experts in health and education systems, do not partner with those working on the creation of AI tools, then we run into allowing AI to drive human decision-making rather than guiding it. Understanding what recommendations we get from AI, how these recommendations were formulated, and whether or not to implement them within the context of global health workforce training is no longer a nice to have task, but a must-have.

## Data Availability

Not applicable.

## Abbreviations

AI: Artificial Intelligence
CoP: Communities of Practice
IT*f*HESE: IT *for* Health and Education System Equity
ML: Machine Learning
TWLG: Technical Working-Learning Group
